# Sudden Hearing Loss and Multiple Cranial Nerve Palsies in Autoimmune disease: A Case Report

**DOI:** 10.22038/ijorl.2020.47096.2608

**Published:** 2021-01

**Authors:** Manisha Narayan, Suja Sreedharan, Rohit Pai, Sajjan Shenoy

**Affiliations:** 1 *Department of Otorhinolaryngology, Kasturba Medical College, Mangalore, Manipal Academy of Higher Education, 575001, Karnataka, India.*; 2 *Department of Neurology, Kasturba Medical College, Mangalore, Manipal Academy of Higher Education, 575001, Karnataka, India.*; 3 *Department of Rheumatology, Kasturba Medical College, Mangalore, Manipal Academy of Higher Education, 575001, Karnataka, India.*

**Keywords:** Multiple cranial nerve palsy, Sudden sensory neural hearing loss, Rheumatoid arthritis

## Abstract

**Introduction:**

The relationship between autoimmune disease and sensorineural loss is well documented in literature. Immune mediated sudden hearing loss is asymmetric, bilateral and rapidly progressive but responds well to steroid therapy. However association of cranial nerve neuropathies with sudden hearing loss is rare.

**Case Report:**

A 41 year old female presented with sudden mixed hearing loss and developed multiple cranial nerve palsies within a month. Blood and Cerebrospinal fluid analysis revealed an undiagnosed rheumatoid arthritis. She responded well to definitive therapy with cyclophosphamide and azathioprine.

**Conclusion:**

If sudden hearing loss is associated with cranial neuropathy, an autoimmune work-up is highly recommended.

## Introduction

The relationship between autoimmune diseases and hearing loss has been well documented. Immune-mediated sensorineural hearing losses (SNHL) occur in women in their thirties and forties; they are usually bilateral, asymmetric and rapidly progressive (1). The temporal progression of autoimmune hearing loss is well recorded; the hearing deteriorates over weeks and months. In 30% of cases, systemic autoimmune disease is associated with the hearing loss, which helps in the diagnosis. Since there are no definitive lab or audiological tests which point towards an autoimmune aetiology, sometimes diagnosis and definitive treatment can be delayed. Positive responses to steroid therapy and relapses on attempts to reduce medication should raise the index of suspicion towards an autoimmune aetiology. Rarely, there is a rapid rise in audiometric thresholds within 72 hours and the patient presents as sudden sensorineural hearing loss (2). The diagnosis is presumed when the hearing rapidly deteriorates in patients with known autoimmune disorders. The hearing loss is bilateral, severe at presentation and does not respond well to therapy (2). Autoimmune disorders can be complicated by neuropathies. The neuropathies involve single or multiple nerves of the peripheral nervous system. Multiple peripheral nerve involvement is associated with or caused by vasculitis; Polyarteritis nodosa, Wegener’s granulomatosis, Churg-Strauss syndrome and immune-mediated diseases like rheumatoid arthritis (RA), systemic lupus erythematosus (SLE), sarcoidosis and cryoglobulinemia (3). Though peripheral nerves are commonly involved, cranial neuropathy has also been described in conditions like Wegener's granulomatosis, polyarteritis nodosa, sarcoidosis and Sjogren's syndrome. However, cranial neuropathies are unusual in RA.

Autoimmune disorders causing both sudden SNHL and multiple cranial nerve palsies are extremely rare. Here we report one such case, and highlight the challenges we faced in the diagnosis and management of the patient who presented with this unusual combination of symptoms.

## Case Report

**Fig 1:**
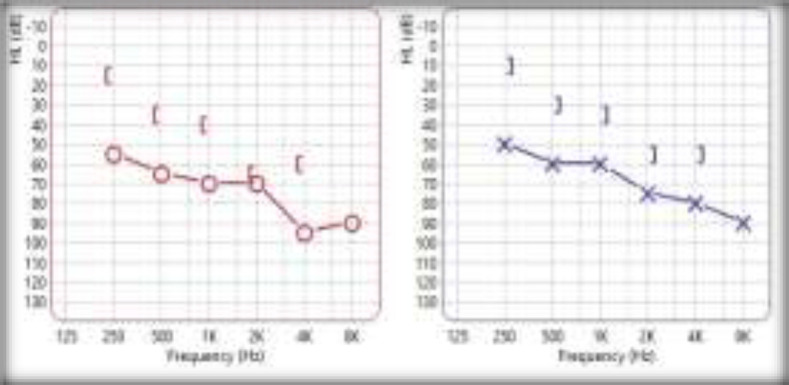
Moderate to profound sloping mixed hearing loss on the right and moderate to severe sloping mixed hearing loss on the left

A 41 years old South Asian female of Indian descent, a homemaker by occupation, presented to the Otorhinolaryngology outpatient department with bilateral hearing loss for one month. The hearing loss was sudden and occurred within two days. The hearing loss was preceded by an episode of upper respiratory tract infection. There was no history of diabetes mellitus, hypertension or tuberculosis. She was taking hormone supplementation for hypothyroidism. There was no relevant family history. On examination of the ear, the tympanic membrane was retracted bilaterally. An audiogram revealed moderate to profound sloping mixed hearing loss on the right and moderate to severe sloping mixed hearing loss on the left with bilateral “B” type tympanogram Fig.1). 

**Fig 2:**
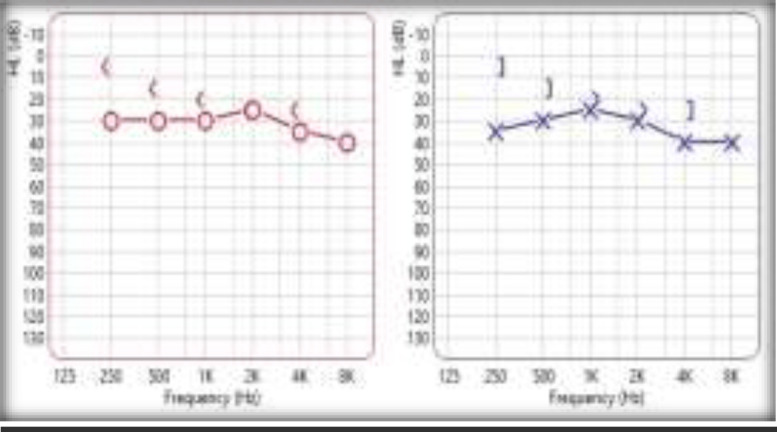
Bilateral mild hearing loss; significant improvement in hearing

Her routine blood investigations were unremarkable except for an erythrocyte sedimentation rate of 42 mm. Ultra-sonogram of the neck showed bulky thyroid gland with nodules in the right lobe. She was treated with systemic steroids (Prednisolone 1mg/kg body weight) for 10 days, which was tapered over 16 days. At the time of discharge, her hearing had dramatically improved (Fig. 2).

Two weeks later, she again presented with decreased hearing in both ears. An audiogram revealed mild to moderately severe mixed hearing loss with bilateral “C” type of tympanogram. On myringotomy, thin serous fluid was present in both middle ears and grommets were inserted bilaterally. Diagnostic nasal endoscopy and blind biopsy of the fossa of Rosenmuller on both sides was also done. Histopathology was reported as normal with no evidence of malignancy. 

After 10 days she presented with facial weakness on the right side and difficulties in speech and swallowing in addition to hearing loss, tinnitus and vertigo. Vertigo was mild, rotatory and brought about by sudden changes in posture. It was not associated with vomiting or sweating. Tinnitus was bilateral, continuous and resembled a whistling tone. Audiology revealed moderate to severe mixed hearing loss on the right and mild SNHL on the left. The impedance on the right side was the ‘Cs’ curve and the ‘A’ curve on the left. She was restarted on steroids (Tab. Prednisolone 1mg/kg body weight). On examination, she had right-sided seventh and left-sided tenth and twelfth cranial nerve palsies. The neurologist opined that there was no peripheral neuropathy. On Functional endoscopic assessment of swallowing there was the presence of pooling of secretions in bilateral pyriform fossae with aspiration. 

On the advice of the Neurologist, autoimmune and paraneoplastic workup was ordered, in addition to radiology and Cerebrospinal fluid (CSF) analysis. Magnetic resonance imaging (MRI) of the brain with contrast showed hyperintensity in bilateral mastoid air cells and middle ear, suggestive of otomastoiditis. There was thickening with diffuse homogenous post-contrast enhancement in posterior and bilateral lateral walls of the nasopharynx. Enhancement was also noted in the 7^th ^and 8^th ^nerve complex on the right side. The CSF analysis was normal with no evidence of malignant cells or paraneoplastic neuronal antibodies.P-Anti-neutrophil cytoplasmic antibody (ANCA) and rheumatoid factor (RF) (31 IU/ml; normal < 18 IU/ml) were reported positive on analysis of blood. Anti-myeloperoxidase (MPO) autoantibodies were reported as highly positive with a value of 10 IU/ml (normal < 5 IU/ml). Anti PR3 autoantibodies were negative. Anti-cyclic citrullinated peptide (anti-CCP) was 50.7 RU/ml (normal < 5 RU/ml). Hence a diagnosis of RA induced mononeuritis multiplex was established. She was given an option of further treatment with cyclophosphamide or rituximab. Due to financial reasons, she opted for treatment with Cyclophosphamide (IV Cyclophosphamide 1g every 3 weeks for 10 doses). The patient tolerated the treatment well and at her last follow up she showed complete recovery from cranial nerve palsies and hearing loss. 

## Discussion

Hughes et al classified inner ear disease associated with autoimmunity into primary and secondary autoimmune inner ear disease (AIED); in the former, the immune response is limited to the ear and in the latter, it is associated with systemic pathologies (4). Association with systemic pathologies is seen in 30% of the cases. Aftab et al. coined the term “immune-mediated inner ear disease” for hearing loss associated with systemic pathology. Malik et al. distinguished between organ-specific diseases termed immune-mediated inner ear disease in which no other systems were involved and systemic immune-mediated inner ear disease with other system involvement (4). This patient had multiple cranial nerve palsies in addition to inner ear disease; however, there was no systemic pathology related to autoimmune disease. Since the final diagnosis was proven to be RA, the term systemic immune-mediated disease seems appropriate. Though there are no special audiological criteria to diagnose AIED, the presence of bilateral SNHL of 30 dB or more at any frequency which shows evidence of progression in at least one ear, at less than three months interval, is considered to be autoimmune inner ear disease (1). It is stressed that the progress of hearing loss is over weeks to months and though fluctuations occur in hearing, there is gradual but steady deterioration in auditory function. Asymmetric bilateral involvement is the norm in audiological evaluations. There may also be associated with vestibular symptoms in 50-80% of patients (1).The patient had a sudden bilateral sensorineural loss, with a conductive component. Sudden SNHL is based on the definition of temporal criteria wherein the bone conduction thresholds increase by at least 30 dB in three continuous frequencies, in few minutes to seventy-two hours. The aetiology is unknown in at least 70-90% of cases (2). Immune-mediated SNHL presenting as the sudden loss is rare. In a study done by Bruno et al evaluation of 337 patients of sudden hearing loss revealed systemic autoimmune disease in 13 (3.83%) patients. Initial audiology in 70% of cases showed severe to profound hearing loss; treatment with steroids did not reverse the hearing loss. Though the hearing loss in our patient resembled sudden sensorineural loss, the improvement of hearing after starting steroids and antimetabolites resembled autoimmune hearing loss which has a slower progression as compared to sudden loss. 

The conductive component in association with sensorineural loss has been demonstrated in a few autoimmune diseases. Polyangiitis causes granulations and effusions, while relapsing polychondritis causes Eustachian tube dysfunction (1). RA can cause conductive hearing loss in several ways. Incudomalleolar and incudostapedial joints can be affected by arthritis leading to increased stiffness. Vasculitis of the blood vessels feeding the incudostapedial joint can cause necrosis of the joint. This patient had a middle ear effusion (proven by myringotomy) and the conductive component disappeared when grommet was inserted into both ears. Eustachian tube dysfunction causing middle ear effusion in RA has not been demonstrated before; this could be due to rheumatoid nodules blocking the eustachian tube. Multiple cranial nerve palsies are known to occur in autoimmune disorders. Autoimmune disorders can cause pachymeningitis affecting multiple cranial nerves in disorders like polyangiitis, RA, Sjogren’s disease, relapsing polychondritis and sarcoidosis. However, the diagnosis is made by thickened dura mater in MRI; this was not seen in our case. Autoimmune vasculitis of small vessels supplying the nerve or inflammation of soft tissue or meninges near a nerve can also cause nerve palsies. Since MRI showed contrast enhancement of 7^th^ and 8^th^ nerve complex on the right side, we assume that it is autoimmune vasculitis that caused cranial nerve palsies in our patient. Cranial nerve palsies are rare in RA; peripheral nerve involvement due to entrapment neuropathies, rheumatoid nodules, swollen synonovium, bony exostosis and inflammatory pannus is common in RA. Rapidly progressive vasculitic neuropathy like the one seen in our patient was the 7th, 8^th^, 10^th^ and 12^th^ nerves were affected in a short period have not been reported before. In this patient, there were no systemic features of an autoimmune disease. On the autoimmune panel, she had a positive anti-cyclic citrullinated peptide (anti-CCP) value with an elevated RF which pointed towards the autoimmune disease, RA. Anti-Myeloperoxidase (MPO) was mildly elevated which ruled out an antineutrophil cytoplasmic antibody-associated (ANCA) vasculitis. Hence, by investigation and clinical presentation, the final diagnosis of mononeuritis multiplex secondary to vasculitis due to RA was made.

## Conclusion

The patient had all features of sudden sensory neural loss, though with a conductive component. With the appearance of multiple cranial nerve palsies in the patient, meningitis with CSF analysis and MRI was ruled out. Workup for systemic pathologies, which led to the diagnosis of RA. Autoimmune disease manifesting as a sudden sensorineural loss with multiple cranial nerve palsies is highly unusual.
